# Predictors of health-related quality of life in patients with irritable bowel syndrome. A cross-sectional study in Norway

**DOI:** 10.1186/s12955-015-0311-8

**Published:** 2015-07-30

**Authors:** Vilde Lehne Michalsen, Per Olav Vandvik, Per G. Farup

**Affiliations:** Department of Research, Innlandet Hospital Trust, N-2381 Brumunddal, Norway; Unit for Applied Clinical Research, Department of Cancer Research and Molecular Medicine, Faculty of Medicine, Norwegian University of Science and Technology, N-7491 Trondheim, Norway; Department of Medicine, Innlandet Hospital Trust, N-2819 Gjøvik, Norway

**Keywords:** Comorbidity, Irritable bowel syndrome, Quality of life, Therapy

## Abstract

**Background:**

Reduced quality of life (QoL) is often the main problem for patients with irritable bowel syndrome (IBS). This study aimed at finding predictors of reduced physical and mental quality of life (QoL) accessible for intervention.

**Methods:**

Consecutive patients with IBS (according to the Rome II criteria) visiting a general practitioner were included in a prospective cohort study and followed up for 6−9 months. At the last visit, information about sociodemographic characteristics, abdominal complaints, QoL and a range of physical and mental comorbidities were collected. Physical and mental QoL were measured with the generic QoL instrument Short Form-12 Physical Component Score (SF-12 PCS) and Short Form-12 Mental Component Score (SF-12 MCS) respectively. The normal scores are 50. This cross-sectional study used data from the last visit.

**Results:**

Out of 208 patients included in the cohort study, 149 (female/male: 105/44) with a mean age of 52 years (SD 15.3) were available for the analyses. Physical and mental QoL were reduced, the mean SF-12 PCS and SF-12 MCS scores were 38.4 (SD 11.9) and 45.0 (SD 11.3) respectively. The main independent predictors of low SF-12 PCS and SF-12 MCS were subjective health complaints and organic diseases, and affective disorders respectively. The severity of IBS symptoms was of minor clinical importance.

**Conclusions:**

To help patients with IBS and reduced QoL, treatment should focus on QoL and not on relief of IBS symptoms. The different causes of reduced physical and mental QoL make an individually directed treatment necessary.

## Introduction

Irritable bowel syndrome (IBS) is a common functional gastrointestinal disorder (FGID). The pooled prevalence is 11.2 % (95 % CI: 9.8 - 12.8 %) with variations between countries from 1.1 to 45.0 %. The variation depends in part on the definition [1, 2]. Although a benign disorder, the symptoms (abdominal pain, bloating, diarrhoea, constipation etc.) are bothersome and a burden for the patients and the society. The disorder is associated with decreased QoL as measured with both generic and disease-specific instruments, a wide range of comorbidities, restrictions of social life and high costs of illness [1, 3–10]. The aetiology and pathogenesis are in part unknown. No cure exists, and the treatment aims at teaching patients to cope with the disorder and to reduce the symptoms with some more or less effective interventions [11–13].

The prevailing assumption is that the gastrointestinal symptoms reduce the QoL and bring about the high costs of illness in patients with IBS. However, a Norwegian study showed that the comorbidity explained most of the high costs and could as well explain the reduced QoL [8]. The reduced QoL and the comorbidity are often more bothersome for patients with IBS than the gastrointestinal symptoms. The IBS severity is associated with reduced generic and disease-specific QoL, but the relative impact of the IBS severity and the variety of comorbidity on generic QoL in general, and on physical and mental QoL in particular, are in large unknown [4, 9, 10, 14–17]. To find ways to improve the care of patients with IBS, this study aimed at finding the clinically most important independent predictors of reduced generic physical and mental QoL accessible for intervention.

## Methods

### Study design and participants

Consecutive patients above 17 years of age consulting 26 Norwegian general practitioners (GPs) at nine health centres during a 10 days’ period in 2001 were asked about abdominal complaints. Patients with abdominal complaints the last three months, for which they had consulted or had wanted to consult a GP, filled in a questionnaire that allowed a diagnosis of IBS according to the Rome II criteria. To exclude other diseases, the GPs performed supplementary examinations at their discretion. Patients with IBS were included in a six months’ follow-up study. At the end of the follow-up period, the participants filled in a structured questionnaire with information about sociodemographic characteristics, abdominal complaints, QoL, and comorbidity. This cross-sectional study used data from the end of the follow-up period. Design details are available in previous publications [3, 8, 18–20].

### Variables

Four groups of variables were collected: Sociodemographic characteristics, IBS symptoms, QoL and comorbidity.

Sociodemographic characteristics: Age (years); gender; daily smoking (yes/no); alcohol use (≤ 2 times per week/> 2 times per week); education (≤ 10 years/> 10 years); and working status (employed, home worker, disability benefit, retired, student, not specified).

IBS symptoms: Symptom duration (years); symptom frequency noted as number of days with symptoms per week (0, 1−2, 3−4, 4−5, >5; scores 0−4); and symptom severity (none, mild, moderate, severe; scores 0−3). IBS symptom intensity score was the product of frequency and severity (range 0−12).

The health-related quality of life was measured with Short Form-12 (SF-12). SF-12 contains eight main elements (general health, physical functioning, bodily pain, role-physical vitality, social functioning, role-emotional, and mental health), and summary scores for physical and mental QoL (SF-12 PCS – physical component score, and SF-12 MCS – mental component score), range 0−100. Only the summary scores were used. The mean summary scores for both SF-12 PCS and SF-12 MCS in the general population are 50 (SD 10) [21].

Comorbidity: Number of organic diseases present the last three months (12 questions about organic diseases, score 0−12). Subjective Health Complaint inventory (SHC) measured complaints the last 30 days. The questionnaire contains 29 questions about common, subjective psychosomatic complaints of which 12 questions were excluded to avoid duplicate assessment of gastroenterological and psychiatric symptoms. The SHC-17 scores subjective somatic complaints (range 0 – 51) with an adjusted population-based mean score of 6.2 (CI 5.9−6.5). Because SHC-17 uses 17 questions out of 29 questions in the original SHC, the adjusted values are 17/29 of the SHC-29 scores [22]. Affective disorders (anxiety and depression) were measured with Hopkins Symptom Check List-10 (SCL-10; score 1.0 – 4.0). Values above 1.85 predict affective disorders [23]. Health anxiety was assessed with Whiteley Index (WI) (score 14−70). The questionnaire has 14 questions related to fear of disease and perception of body and health; values > 40 indicate hypochondria [24]. Neuroticism was assessed with a short form of Eysenck Personality Questionnaire (EPQ); score 0−10 [25, 26].

### Statistics

Depending on the distribution of the data (tested with the Kolmogorov-Smirnov and Shapiro-Wilk) associations between QoL and other variables were analysed with t-test and one-way ANOVA, and correlations with Pearson and Spearman correlation tests. Missing data in covariates were handled by multiple imputation. All variables used in the regression analyses were used in the imputation model. M = 100 imputed data sets were created as recommended by van Buuren [27]. The dependent variables were included as predictors in the imputation model but were not imputed. The pooled estimate with CI and *p*-values were obtained using Rubin’s rules for multiple imputations. Multiple imputation is a recommended method to include subjects with partially missing data on covariates in the analyses. All subjects with complete data on the dependent variable, and with complete or partially missing data on covariates, are included in the analysis.

To detect predictors of SF-12 PCS and SF-12 MCS, the first set of linear regression analyses included all sociodemographic characteristics, IBS duration and IBS symptom intensity score and one at the time of each of the five comorbidities in five consecutive analyses. The final set of regression analyses performed to detect independent predictors of SF-12 PCS and SF-12 MCS included all sociodemographic variables, IBS duration, IBS symptom intensity score, and all the comorbidities that were significantly associated with SF-12 PCS and SF-12 MCS in the first set of regression analyses. The results are given as the constants, regression coefficients (B) with 95 % confidence intervals (CI), partial correlations (pc) and *p*-values. The data were analysed with SPSS version 20.

### Ethics

The study conformed to the principles of the Declaration of Helsinki and was approved by the Norwegian Regional Committees for Medical and Health Research Ethics. Before inclusion in the study, all patients gave written informed consent.

## Results

### Participants

**Fig. 1:**
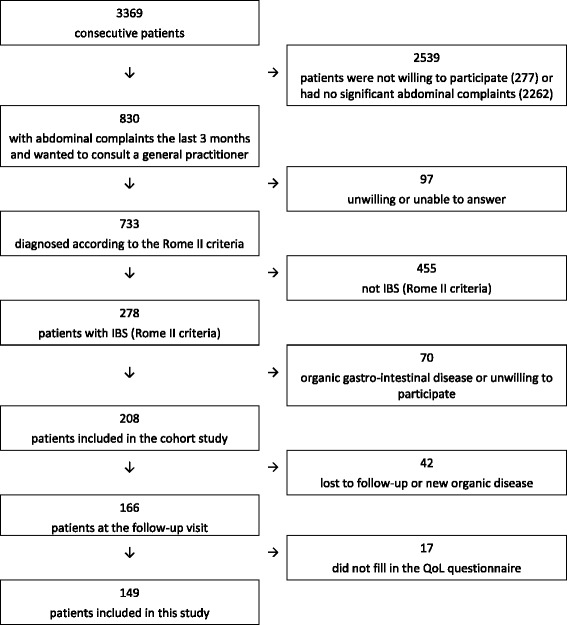
Flow chart of the patients in the study

830 out of 3369 consecutive patients reported abdominal complaints the last three months. 278 had IBS according to the Rome II criteria, of whom 208 were included in the follow-up study. 149 patients (105 females and 44 men, mean age 52 years) with information about QoL were available for analyses 6−9 months after inclusion. Figure 1 shows a flow chart of the patients with a detailed account for all exclusions, and Table 1 gives the characteristics of the patients.

**Table 1:** Patient characteristics

Characteristics (no of patients)	Results
Female/male	105 (70 %)/44 (30 %)
Age (years) (*n* = 148)	52.0 (15,3)^a^
Education (< 10 years/10−13 years/> 13 years)	52 (37 %)/57(41 %)/31(22 %)
Daily smokers (*n* = 138)	49 (36 %)
Alcohol use (> 2 times per week) (*n* = 135)	23 (17 %)
Employment status (*n* = 140)	
Employed	66 (47 %)
Disability benefit	27 (19 %)
Home worker	8 (6 %)
Student	7 (5 %)
Retired	29 (21 %)
Not specified	3 (2 %)
Duration of IBS (Years) (*n* = 133)	10 (1−55)”
IBS symptom intensity score (score 0−12)	2.0 (0−12)”
Number of organic diseases	2 (0−9)”
Whiteley Index (WI) (*n* = 139)	25.0 (14.0−60.0)”
Hypochondria (WI > 40)	9 (6 %)
Hopkins Symptom Check List-10 (SCL-10) (*n* = 141)	1.7 (1.0−3.8)”
Affective disorder (SCL-10 > 1.85)	57 (40 %)
Eysenck Personality Questionnaire (*n* = 142)	4.0 (0−10.0)”
Subjective Health Complaints-17 (*n* = 148)	12.0 (0−42)”
SF-12 Physical Component Scale	38.4 (11.9)^a^
SF-12 Mental Component Scale	45.0 (11.3)^a^

The number of patients available for the analysis is given in brackets (*n* = x) if less than *n* = 149

Results are given as numbers with percentages, mean with SD (^a^) or median with range (”)

### QoL

Mean SF-12 PCS and SF-12 MCS were 38.4 (SD 11.9) and 45.0 (SD 11.3) respectively. SF-12 PCS and SF-12 MCS were negatively associated with IBS symptom intensity score, SHC-17, WI and SCL-10. SF-12 PCS was also negatively associated with the number of organic diseases and short education, and SF-12 MCS was negatively associated with EPQ and smoking. Table 2 gives all associations between the QoL and the patient characteristics.

**Table 2:** Associations between patient characteristics and the physical (SF-12 PCS) and mental (SF-12 MCS) components of QoL

Patient characteristics (no of patients)	SF-12 PCS	*p*-value	SF-12 MCS	*p*-value
Age (*n* = 148)	*r* = -0.212	0.010	*r* = 0.259	0.002
Gender (male/female)	38.9 (12.3)/38.2 (11.8)	0.736^a^	47.4 (11.3)/44.0 (11.1)	0.091^a^
Education (< 10 years/10-13 years/> 13 years)	36.8 (11.3)/38.5(12.7)/42.8(11.0)	0.037”	44.5 (10.8)/45.0(11.7)/44.4(10.7)	0.955”
Daily smoking (yes/no) (*n* = 138)	38.7 (11.9)/38.6 (12.2)	0.976^a^	42.0 (9.9)/46.4 (11.5)	0.025^a^
Alcohol (≤ 2/week/> 2/week) (*n* = 135)	37.6 (12.2)/41.9 (11.9)	0.119^a^	45.0 (10.8)/44.2 (12.9)	0.754^a^
IBS symptom intensity score	rho = -0.232	0.004	rho = -0.229	0.005
Duration of IBS (*n* = 133)	rho = -0.033	0.705	*r* = 0.017	0.844
Subjective Health Complaints-17 (*n* = 148)	rho = -0.518	0.000	rho = -0.283	0.001
Eysenck Personality Questionnaire (*n* = 142)	rho = -0.024	0.778	rho = -0.616	< 0.001
Whiteley Index (*n* = 139)	rho = -0.395	< 0.001	rho = -0.441	< 0.001
No. organic diseases	rho = -0.407	< 0.001	rho = 0.000	0.995
Hopkins Symptom Check List-10 (*n* = 141)	rho = -0.191	0.024	rho = -0.695	< 0.001

The number of patients available for the analysis is given in brackets (*n* = x) if less than *n* = 149

SF-12 PCS = Short Form 12 Physical Component Score

SF-12 MCS = Short Form 12 Mental Component Score

The results are given as mean (SD) or correlations with *r*-value (Pearson) or rho-value (Spearman)

^a^t-test, “ One-way ANOVA

### Independent predictors of QoL

In the first set of regression analyses, SF-12 PCS was statistically significantly associated with SHC-17 (pc = -0.470, *p* < 0.001), number of organic disease (pc = -0.365, *p* < 0.001) and WI (pc = -0.331, *p* < 0.001); and with IBS symptom intensity score when adjusting for number of organic diseases and EPQ. SF-12 MCS was statistically significantly associated with SCL-10 (pc = -0.656, *p* < 0.001), EPQ (pc = -0.567, *p* < 0.001), WI (pc = -0.441, *p* < 0.001) and SHC-17 (pc = -0.182, *p* = 0.015); and with IBS symptom intensity score except when adjusted for SCL-10. IBS duration was not statistically significantly associated with either SF-12 PCS or SF-12 MCS.

**Fig. 2:**
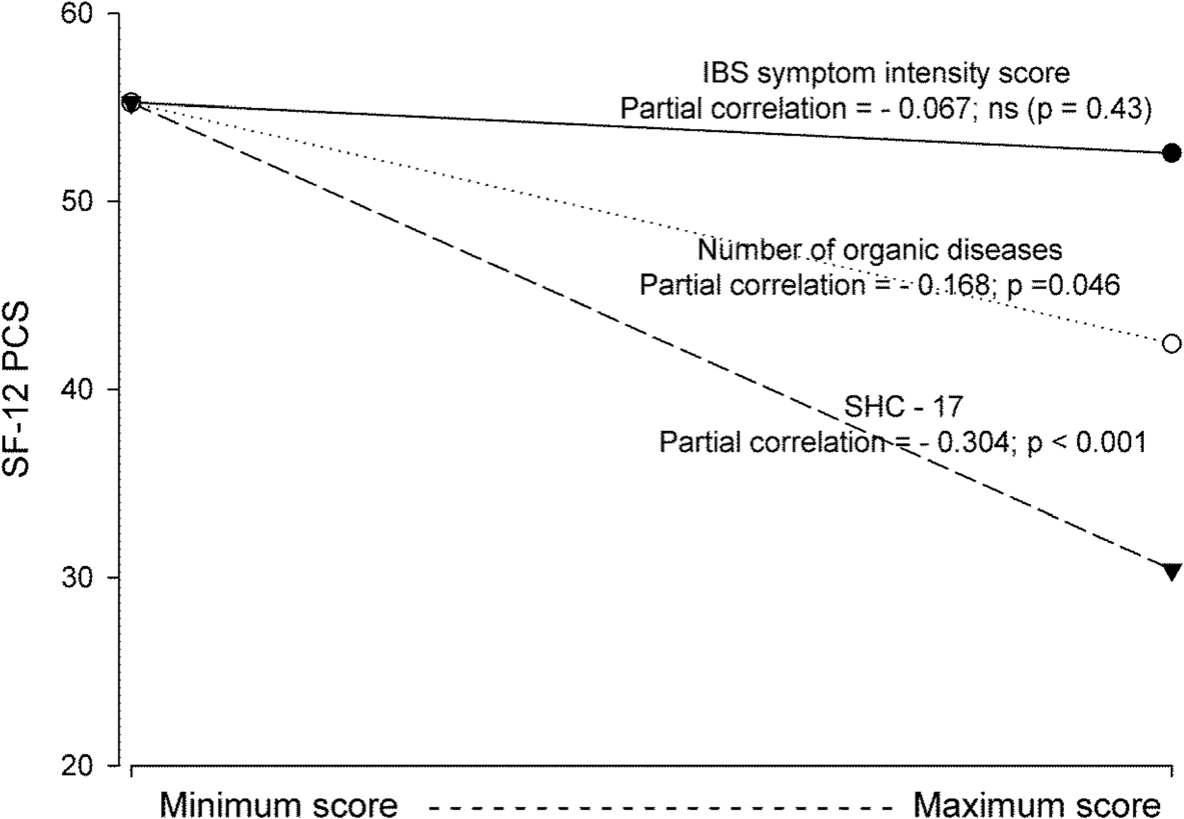
The associations between physical QoL (SF-12 PCS) and IBS symptom intensity score, number of organic diseases and Subjective Health Complaints-17 (SHC-17). Presentation of the results from the linear regression analyses

**Fig. 3:**
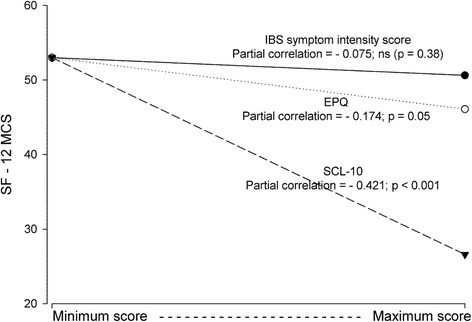
The associations between mental QoL (SF-12 MCS) and IBS symptom intensity score, Eysenck Personality Questionnaire (EPQ) and Hopkins Symptom Check List-10 (SCL-10). Presentation of the results from the linear regression analyses

The Tables 3 and 4 give the results of the final set of regression analyses. Independent predictors were sociodemographic characteristics, IBS duration and IBS symptom intensity score, and, in addition, the comorbidities significantly associated with SF-12 PCS or SF-12 MCS in the first set of regression analyses. Neither IBS duration nor IBS symptom intensity score was statistically significantly associated with QoL. The strongest independent predictors for SF-12 PCS and SF-12 MCS were SHC-17 (pc = -0,304; *p* < 0.001) and SCL-10 (pc = -0,421; *p* < 0.001) respectively. The Figs. 2 and 3 show graphic presentations of the most important results.

**Table 3:** Predictors of physical QoL (SF-12 PCS) when adjusting for sociodemographic variables, IBS variables, Subjective Health Complaints-17, Whiteley Index and number of organic diseases (linear regression analyses with all the reported variables included in the analysis)

Variable	*p*-value	Partial correlation (pc)	Regression coefficient B (95 % CI)
Constant			54.25 (47.05; 61.46)
Age (years)	0.050	−0.167	−0.13 (-0.26; -0.00)
Male gender	0.896	−0.011	−0.25 (-3.95; 3.45)
Daily smoking	0.711	−0.032	−0.71 (-4.46; 3.04)
Alcohol more than 2/week	0.306	0.090	2.45 (-2.25; 7.15)
Education (< 10 years/10-13 years/> 10 years)	0.810	0.021	0.31 (-2.25; 2.87)
IBS duration (years)	0.721	0.032	0.02 (-0.09; 0.14)
IBS symptom intensity score	0.434	−0.067	−0.22 (-0.78; 0.34)
Subjective Health Complaints-17	< 0.001	−0.304	−0.49 (-0.74; -0.23)
Whiteley Index	0.090	−0.146	−0.20 (-0.43; 0.03)
Organic disease (number)	0.046	−0.168	−1.07 (-2.12; -0.02)

**Table 4:** Predictors for SF-12 MCS when adjusting for sociodemographic variables, IBS variables, Subjective Health Complaints-17, Whiteley Index, Hopkins Symptom Check List-10 and Eysenck Personality Questionnaire (linear regression analyses with all the reported variables included in the analysis)

Variable	*p*-value	Partial correlation (pc)	Regression coefficient B (95 % CI)
Constant			52.63 (46.81; 58.45)
Age (years)	.013	0.210	0.12 (0.03; 0.21)
Male gender	.879	0.013	0.23 (-2.73; 3.18)
Daily smoking	.302	−0.091	−1.53 (-4.44; 1.37)
Alcohol > 2/week	.862	−0.015	−0.32 (-3.97; 3.33)
Education (<10 years/10-13 years/>10 years)	.725	−0.031	−0.36 (-2.38; 1.65)
IBS duration (years)	.715	0.034	0.02 (-0.08; 0.11)
IBS symptom intensity score	.379	−0.075	−0.20 (-0.64; 0.24)
Subjective Health Complaints-17	.448	0.066	0.07 (-0.12; 0.26)
Whiteley Index	.153	−0.127	−0.15 (-0.37; 0.06)
Hopkins Symptom Check List-10	< 0.001	−0.421	−8.80 (-12.08; -5.53)
Eysenck Personality Questionnaire	.050	−0.174	−0.69 (-1.38; -0.00)

## Discussion

In this study, the comorbidity was the strongest predictor of reduced general QoL in patients with IBS. Neither IBS duration nor IBS symptom intensity score was of clinical significance. These findings have important implications for the handling of the patients since reduced QoL often is the main clinical problem.

In this group of patients with IBS recruited in general practice, both physical and mental QoL were reduced, the physical component apparently more than the mental one. Since most studies report reduced QoL in patients with IBS, these findings were anticipated [9, 10, 14, 15]. Patients recruited in the community will probably have less affection of QoL, and patients in secondary and tertiary centres more reduced QoL than in primary care [14]. The reasons for consultations for IBS vary [28]. In this study, the comorbidity and reduced QoL, and not the IBS symptoms, might have initiated the consultation since the IBS duration was long (median 10 years) and the IBS symptom intensity score was low (median score 2). Physical and mental QoL were normal (approximately 50) if the characteristics of the patients were normalised (the constants in the Tables 3 and 4), which indicate that the most important factors affecting QoL were measured.

The aetiology of reduced generic QoL is complex and varies between disorders. In one study of patients with ulcerative colitis and IBS, the most important predictors of reduced QoL were the pain severity and the catastrophization respectively [29]. Organic diseases, unexplained somatic health complaints, affective psychiatric disorders (anxiety and depression), cognitive dysfunction, major psychiatric disorders (e.g. psychosis) and social dysfunction all affect QoL. Since IBS has been associated with most of these complaints and, therefore, has been classified as a bio-psycho-social disorder, reduced QoL is expected.

In this study, the prevalence of comorbidity was high. The prevalence of subjective health complaints and affective disorders were twice and nearly four times those in the general Norwegian population, and 6 % of the patients had WI scores indicating hypochondria [22, 23]. In large, the prevalence of comorbidity was in accordance with previous reports in patients with IBS. However, only a few studies take into account other organic diseases and subjective health complaints, and comparative studies with the same diagnostic tools are unavailable [4, 14, 16]. Social functions were not studied.

The combination of IBS, a variety of unexplained somatic symptoms, organic diseases, psychological affection and reduced QoL is common. These striking associations raise the question whether IBS is one distinct disorder, several distinct disorders, or only one out of many symptoms in a generalised systemic disorder. For the treatment of the patients, these considerations are of limited interest since no treatment is available for all patients with IBS. Treatment should focus on the patients’ main symptom(s). In patients in whom reduced overall QoL is the main symptom, improvement of QoL should be the primary aim. The treatment of QoL should be individualised and directed against the cause of reduced QoL, which varies [14, 16, 29]. To individualise the treatment, knowledge about the cause of reduced QoL is mandatory.

This study showed several highly significant associations between symptoms accessible for intervention and generic QoL. IBS symptom intensity score, subjective health complaints, and affective disorders and health anxiety were associated with reduced physical and mental QoL. In addition, the number of organic diseases was associated with reduced physical QoL, and neuroticism with reduced mental QoL. Since the patients present with abdominal symptoms and the symptoms strongly correlate with QoL, the treatment usually aims at reducing the abdominal symptoms. The wide range of symptoms was, however, highly associated with each other and made analyses of independent predictors necessary. Analyses of independent predictors including organic diseases and subjective health complaints have, to our knowledge, not been performed [4, 16, 29]. In the analyses of independent predictors of QoL, subjective health complaints and to a lesser extent organic diseases were independent predictors of reduced physical QoL, and affective disorders and neuroticism predicted reduced mental QoL. It is notable that the cause of reduced physical and mental QoL differed and that IBS symptom intensity score did not predict either physical or mental QoL after adjusting for the comorbidity.

The findings have important clinical implications. The care of patients with IBS should focus on the main symptoms. If the main symptom is reduced physical QoL, the care should focus on relief of subjective health complaints other than gastrointestinal symptoms (e.g. muscle-skeletal disorders) and on organic disorders; and on affective disorders (e.g. anxiety and depression) in patients with reduced mental QoL [30]. To our knowledge, the nonsignificant clinical effect of IBS symptoms on QoL, the inclusion of organic diseases and subjective health complaints in the evaluations of QoL in patients with IBS, and the distinction between physical and mental QoL have not previously been reported. An individually tailored treatment depending on the type and cause of reduced QoL should be aimed at in patients with IBS and reduced QoL, and not relief of IBS symptoms.

### Strengths and limitations

The inclusion of consecutive patients with abdominal complaints visiting GPs, the high participation rate (92 %) and the accordance with other studies indicate a satisfactory external validity [31]. The follow-up visit was planned six months after inclusion, but for a minority the visit took place as late as nine months after inclusion for practical reasons. In this cross-sectional study, the patients were asked to report symptoms from the 6−9 months’ study period. Since IBS is an undulating disorder, recordings from another period could have modified the results.

The patients were older than in most other studies. Age has in most studies a limited effect on QoL in patients with IBS [30, 32]. In contrast to most other studies, this study takes into account the somatic comorbidity, which is a strength and of importance for the evaluation of QoL. Patients with somatic comorbidity might even have been excluded from other studies. It is unlikely that the organic disorders caused the IBS symptoms.

The diagnosis of IBS and the measurement of QoL and the comorbidities were performed with acknowledged instruments validated in Norwegian, and the range of comorbidities taken into account was high. The use of a generic QoL questionnaire like SF-12 was judged as a better overall measure of the patients’ overall QoL than a disease-specific tool. The impact of IBS symptom severity is probably higher on the IBS specific QoL than on the general QoL. A generic QoL instrument was used since the study aimed at finding ways to improve the patients’ general QoL. Studies and physicians often focus on the disorder and the disease-specific QoL and forget the patients’ overall complaints. The use of both a generic and disease-specific QoL questionnaire would have been preferable. It is likely that the most important variables influencing QoL were registered since the physical and mental QoL were normal (values approximately 50) in patients with low scores for IBS symptom intensity and no comorbidities, (i.e. the constants in the multivariable analyses were approximately 50). In the final dataset used for the multivariable analyses, multiple imputations of missing data were performed. In this study, there were different predictors of physical and mental QOL, a finding that deserves attention in clinical practice.

Valid information about current management of the patients was not available, and ongoing treatment might have influenced the QoL. The patients were for sure managed as well as possible according to the GPs’ assessment, and the results give an overall estimate of the QoL and factors affecting the QoL in patients with IBS consulting their GP.

The Rome II criteria, and not the new Rome III criteria, were used in the study. A possible misclassification of the patients due to the use of the Rome II criteria instead of the Rome III criteria was judged as insignificant. The previously observed poor agreement between the GPs’ diagnosis of IBS and the Rome criteria reduces the validity for the use of the results in general practice [19]. The IBS symptom intensity score used in this study is not a validated tool but has been used in several studies [1, 3]. It was chosen because it has been used by the Norwegian Health Authorities in the national health surveys. A validated severity score like “The irritable bowel severity scoring system” would have been preferable [33]. The participation rate in the first part of the study was high, but dropouts and missing data at the final visit were significant and might have reduced the external validity. Only 149 out of 278 patients (54 %) with IBS were available for the analyses. Data “missing not at random” (e.g. dropout of patients with low or good QoL) might have reduced the internal validity [34]. In this study, most patients had a long duration of IBS. In patients with new onset of the disease, the results might be different.

Finally, a cross-sectional study only shows associations and not causality. Nevertheless, until causality has been shown in other studies, this information should be used in the handling of patients with IBS and reduced QoL.

## Conclusions

The study indicated that treatment of patients with IBS and low physical QoL should focus primarily on subjective health complaints and organic diseases, and in patients with low mental QoL on affective disorders. The severity of IBS symptoms was of minor clinical importance for the physical and mental QoL.
